# Validity and reliability of the Persian version of Recce stigma scale in people with multiple sclerosis and its impact on quality of life

**DOI:** 10.1186/s12883-024-03544-z

**Published:** 2024-01-22

**Authors:** Mohammad Amin Habibi, Mohammad Yazdan Panah, Saeed Vaheb, Meysam Olfatifar, Aysa Shaygannejad, Yousef Mokary, Majid Ghasemi, Sangharsha Thapa, Vahid Shaygannejad, Omid Mirmosayyeb

**Affiliations:** 1 Multiple Sclerosis Research Center, Neuroscience Institute, Tehran University of Medical Sciences, Tehran, Iran; 2 Students Research Committee, Shahrekord University of Medical Sciences, Shahrekord, Iran; 3 Isfahan Neurosciences Research Center, Isfahan University of Medical Sciences, Isfahan, Iran; 4 Gastroenterology and Hepatology Diseases Research Center, Qom University of Medical Sciences, Qom, Iran; 5 Department of Neurology, School of Medicine, Isfahan University of Medical Sciences, Isfahan, Iran; 6 Westchester Medical Center, New York Medical College, Valhalla, NY USA

**Keywords:** Multiple sclerosis, Social Stigma, Validity, Reliability, Persian

## Abstract

**Background:**

There is often a fear of social stigma experienced by people with multiple sclerosis (pwMS), which negatively impacts the quality of their lives (QoL). Currently, no Persian-validated questionnaire is available to assess this issue in pwMS. This study aimed to assess the validaty and reliability of the Persian version of Reece Stigma Scale Multiple Sclerosis (RSS-MS) questionnaire for pwMS.

**Method:**

This cross-sectional was conducted between January and February 2023 in Isfahan, Iran. The demographic and clinical information and the RSS-MS and Multiple Sclerosis Impact Scale-29 (MSIS-29) questionnaires were recorded from pwMS. The content validity index (CVI) and content validity ratio (CVR) have been used to evaluate validity. To identify the factors supporting the MS-related stigma, an exploratory factor analysis (EFA) was conducted.

**Results:**

The present study recruited 194 pwMS. Based on factor analysis, only two factors had eigenvalues ≥ 1.0 and exhibited high internal consistency. The Cronbach’s α coefficient for internal consistency of the RSS-MS scale was 0.822. More evidence for the construct validity suggested that having higher levels of stigma is significantly correlated with psychological (r = 0.468, *p-value* < 0.001) and physical dimensions (r = 0.585, *p-value* < 0.001) of MSIS-29. Expanded Disability Status Scale, disease duration, and treatment duration did not show a significant correlation with stigma (*p-value* > 0.05).

**Conclusion:**

This study indicated that the modified version of the RSS-MS scale in the Persian language showed acceptable validity and reliability for evaluating the stigma among Persian pwMS. Furthermore, this study emphasizes the cruciality of monitoring and addressing stigma among pwMS, as it can potentially enhance medical, psychological, physical, and QoL outcomes.

**Supplementary Information:**

The online version contains supplementary material available at 10.1186/s12883-024-03544-z.

## Introduction

Multiple sclerosis (MS) is a long-term autoimmune disorder characterized by its impact on the central nervous system (CNS) and the potential to cause different forms of disabilities that pose substantial challenges to individuals, medical professionals, and governing bodies [1]. Recent statistics from the Global Burden Disease (GBD) report in 2016 show that the prevalence of MS has increased by 10.4% worldwide between 1990 and 2016, highlighting the growing socio-economic impact of MS over the past few decades [2].

Stigma denotes the unfavorable sentiments of the general public towards an individual’s condition or circumstances [3]. Stigma can be a problem found in patients with chronic diseases, particularly neurological disorders [4]. Stigma is allegedly divided into public and self-stigma: Public stigma is labeling patients with unwelcome features because of the prevailing and cultural type, which can result in self-stigma. Studies have shown that stigma affects between 20% and 80% of patients with relapsing-remitting MS and primary-progressive MS, respectively [5, 6]. This indicates that stigma is a significant issue for those with MS, who already experience disability and reduced work productivity [7]. Disability and stigma are closely related, as patients with higher physical disability experience a poorer quality of life (QoL) and lower work productivity [8, 9]. Unfortunately, society judges people with multiple sclerosis (pwMS) based on their clinical conditions, which can result in psychological stress, such as depression, loss of confidence, and difficulties finding employment or forming relationships [10]. Additionally, the sense of stigmatization in pwMS is an obstructing factor for the adherence of patients to medical management and intensive follow-up. Therefore, determining the level of stigmatization in pwMS is highly associated with clinical benefits.

Several stigma scales have been developed to assess stigma associated with chronic illnesses, including the Chronic Illness Anticipated Stigma Scale [4], the Stigma Scale for Chronic Illness [11], and the Internalized Stigma of Mental Illness scale [12]. Regarding MS, the Reece Stigma Scale Multiple Sclerosis (RSS-MS) questionnaire was designed by Eldridge-Smith et al. [13] to examine the effects of social stigma and fear in pwMS and to understand the importance of stigma in their lives. The reliability and validity of this questionnaire were assessed in Korean [3] and Turkish [14] languages, but no study has examined the reliability and validity in the Persian language among people with multiple sclerosis (pwMS). While this questionnaire has been recently validated for pwMS, more studies are needed to explore the perception of stigma among pwMS.

This study aimed to assess the validity and reliability of the Persian translation of the RSS-MS and to evaluate its impact on QoL outcomes among Persian pwMS.

## Method

### Ethical statement

All patients provided written informed consent after being informed of the study protocol. The study was approved by the Ethics Committee of Isfahan University of Medical Sciences (IR.MUI.MED.REC.1400.351).

### Patients

This cross-sectional study was conducted from January to February 2023 on pwMS referred to the Kashani MS clinic in Isfahan, Iran. Eligible patients were considered to have the following criteria: (1) diagnosis of MS according to McDonald criteria [15], (2) patients aged between 18 and 60 years old, and (3) having a disease duration of 6 months to 10 years. Patients who were unwilling to participate were excluded from our study.

### Translation

RSS-MS validity and reliability for pwMS was previously established [13]. The forward-backward method was adopted for the RSS-MS translation from English into Persian (the Iranian language). Two general practitioners translated the questionnaire into Persian. Following this, a health professional and professional translator translated the document backward into English. Subsequently, an initial edition of the Iranian survey was provided. The final version was developed after all authors agreed on the cultural adaptations of some problematic terms (Supplementary, Table S1).

### Outcome measures and Procedure

A three-sect checklist was provided to collect demographic and clinical information. In the initial section of the checklist, the patient’s demographic and clinical details, including their name, age, weight, height, disease duration, Expanded Disability Status Scale (EDSS) [16], and disease-modifying therapies (DMTs), were collected. The second section of the checklist pertained to the Persian-translated version of RSS-MS questionnaire. The Persian version of RSS-MS questionnaire contains nine questions and is answered in the spectrum of “never,” “rarely,” “sometimes,” “often,” and “always” on a 5-point scale of never:1 to always:5. In the third part of the checklist, individuals were requested to respond to questions in a booklet incorporating the Persian version of Multiple Sclerosis Impact Scale-29 (MSIS-29) questionnaire [17] to control the effect of MS disorder, as a cofounder variable, on our results. The MSIS-29 is a scale containing 29 questions answered with “not at all,” “a little,” “moderate,” “a lot,” and “very much” and scored from 1 to 5, respectively, divided into two subcategories, a 20-item scale assessing physical influence (MSIS-29-PHYS) and a 9-item scale measuring psychological influence (MSIS-29-PSYCH). All items utilize a polytomous response format with a range of 1–5, where higher scores indicate greater impact levels. The total score for each subcategory can be calculated by combining item scores and converting them into a score out of 100. Patients with a cumulative score of over 80 were associated with high quality [18].

### Measure adaptation

To determine the validity of the Persian version of RSS-MS questionnaire, researchers followed Downing’s multi-component approach, which matched our goals for the data at the beginning and end stages. Downing’s method involves five components: content, internal structure, relationship to other variables, response process, and consequences, which are used to verify measurements. This framework has been used to confirm the validity of various measures, including medical resident communication and interpersonal skills, medical skills assessment, effective clinical teaching methods, and crisis management self-efficacy. The inter-rater expert agreement was assessed using the content validity coefficient to assess the content validity.

### Validity assessment

We determined the content validity of the Persian version of RSS-MS questionnaire by assessing the content validity index (CVI) and the content validity ratio (CVR) indices for every item of the questionnaire, utilizing a panel of ten experts. Then, we checked the construct validity by applying the exploratory factor analysis (EFA) to establish the factor structure that endorses the MS-related stigma recognized by healthcare professionals. The maximum likelihood (ML) method was applied to retrieve the loading factors of questionnaire items. A factor loading is determined by calculating the correlation between the factor and an item. When the factor loading is higher than 0.30, it generally means a moderate correlation between the item and the factor. We conducted a thorough examination to assess the optimal number of potential factors. We strictly evaluated their adherence to the Kaiser-Meyer-Olkin (KMO) criteria of eigenvalues ≥ 1, and the variance ratio was described by each factor [19].

We employed the KMO test to verify sampling sufficiency [20]. The KMO is a metric that represents the degree to which latent factors may influence the variability of your variables. A higher value, closer to 1.0, may suggest that a factor analysis could be helpful. However, the outcomes of the factor analysis may be unproductive if the value is less than 0.50.

Bartlett’s sphericity test considers the correlation matrix and checks whether the variables are suitable for structure detection. Factor analysis would be appropriate for analyzing your data if the significance level is less than 0.05.

### Reliability analysis

To check the reliability of the Persian version of RSS-MS questionnaire, Cronbach’s coefficient alpha was used to ascertain the consistency of the items and the correlations between them. The sample size of our study for reliability assessment was appropriately chosen based on existing literature [21–23].

### Statistical analysis

The unidentifiable data of patients were inserted into a pre-designed Excel sheet. The IBM SPSS Statistics for Windows, version 26 (IBM Corp., Armonk, NY, USA) was employed to conduct the data analysis. The Kolmogorov-Smirnov test checked the normality of variables. Parametric tests and others with non-parametric tests analyze the variables with normal distribution. Continuous data was shown via mean ± standard deviation (SD), and categorical data were demonstrated via absolute numbers and percentages. The independent sample t-test, Mann-Whitney U, and Chi-squared tests were used for continuous and categorical variables, respectively. A one-way ANOVA test was also conducted between stigma, EDSS, MSIS-29-PSYCH, and MSIS-29-PHYS scores. The correlation between subclasses of stigma was also calculated. The relationships between the RSS-MS and instruments of constructs related to stigma were assessed using Pearson correlations and mean testing to provide additional validation information. Statistics were considered significant at a *p-value* < 0.05.

## Results

### Baseline characteristics

Our study consisted of 194 (73.7% were female) pwMS with a mean (SD) EDSS score of 1.23 ± 1.49. The mean (SD) disease and treatment duration were 8.98 ± 5.68 and 3.44 ± 3.11 years, respectively. The demographic and clinical characteristics of participants are represented in Table 1.

**Table 1:** Baseline demographic and clinical characteristics

Variables	Value
Age, (Mean ± SD) (years)	37.88 ± 9.2
Gender, (n, %)	
Male	51 (26.3%)
Female	143 (73.7%)
Marital state, (n, %)	
Single	51 (26.3%)
Married	134 (69.1%)
Divorced	9 (4.6%)
Education, (n, %)	
Elementary	5 (2.6%)
Cycle	20 (10.3%)
Diploma	76 (39.2%)
Bachelor	70 (36.1%)
Upper than bachelor	23 (11.9%)
Job state, (n, %)	
Employed	93 (47.9%)
Unemployed	101 (52.1%)
Disease duration (Mean ± SD) (years)	8.98 ± 5.68
Treatment duration (Mean ± SD) (years)	3.44 ± 3.11
EDSS score, (Mean ± SD)	1.23 ± 1.49
Treatment, (n, %)	
IFN-B1a	33 (17%)
IFN-B1b	8 (4.1%)
Rituximab	68 (35.1%)
Ocrelizumab	4 (2.1%)
Glatiramer acetate	4 (2.1%)
Fingolimod	13 (6.7%)
Teriflunomid	32 (16.5%)
DMF	18 (9.3%)
Fampridine	4 (2.1%)
No treatment	10 (5.2%)

DMF: Dimethyl fumarate, EDSS: Expanded Disability Status Scale, IFN: Interferon

### Reliability and validity assessments

Table 2 demonstrates the factor loading of items. KMO and Bartlett’s tests were used to determine the effectiveness of factor analysis. The ML method was used to perform EFA on all nine items. The KMO index of sampling adequacy was 0.848, indicating that factor analysis would yield useful results. Additionally, Bartlett’s test *p-value* < 0.001 indicated a significant difference between the variance of items. Based on EFA analysis, all questionnaire items were qualified for the RSS-MS stigma questionnaire in Iranian patients. Accordingly, factor loading and eigenvalues of all items were at an acceptable level. Two factors had an eigenvalue ≥ 1.0, while seven items had an eigenvalue < 0.5. Moreover, our model had more acceptable sampling accuracy with coefficient of 0.822. The inter-item relation was investigated and ranged from 0.387 to 0.618. The result was not significantly improved after removing items with low-total correlations and recalculating Cronbach’s coefficients.

**Table 2:** Item loading and eigenvalue for RSS-MS Questionnaire

Items	Factor Loading	CVI	CVR
1. I felt that having MS was a punishment for things I had done.	3.867	1	1
2. Felt that people were avoiding me because of my MS.	1.005	1	1
3. I am afraid that I would lose my friends if they knew about having MS.	0.963	1	1
4. Felt like people that I know were treating me differently because of my MS.	0.686	1	1
5. Felt like people look down on me because I have MS.	0.646	1	1
6. Avoided dating because most people don’t want a relationship with someone with MS.	0.597	1	1
7. I Avoided a situation because I was worried about people knowing I had MS.	0.491	1	1
8. I was embarrassed about having MS.	0.397	1	1
9. Felt that keeping my MS a secret was important.	0.348	1	1

CVI: content validity index, CVR: Content Validity ratio, RSS-MS: Reece Stigma Scale-Multiple Sclerosis

### Association between demographic and clinical characteristics and stigma

Table 3 shows no significant differences in stigma, MSIS-29-PSYCH, and MSIS-29-PHYS scores between patients with different demographic and clinical characteristics (*p-value* > 0.05). A significant difference was observed in MSIS-29-PHYS scores between unemployed (51.6 ± 8.76) and employed patients (55.66 ± 10.31) (*p-value* = 0.03).

**Table 3:** Relationships between demographic, clinical characteristics, and stigma

Variables (Mean±SD)	Stigma	*p*-value	Psychological effects	*p*-value	Physical effects	*p*-value
Sex		0.608		0.076		0.286
Female	26.24±3.79		22.16±4.86		53.1±9.14	
Male	26.56±4.06		23.66±6.00		54.8±11.22	
Marital		0.137		0.814		0.610
Single	25.49±3.9		22.15±5.94		52.39±10.24	
Married	26.55±3.8		22.7±4.94		53.94±9.33	
Divorced	27.66±3.93		22.55±5.17		54.33±13.02	
Education		0.353		0.175		0.465
Elementary	28.2±4.81		23.2±4.91		54.2±10.08	
Cycle	26.65±3.46		24.85±5.47		52.5±12.58	
Diploma	25.68±3.79		21.94±5.16		52.9±9.5	
Bachelor	26.78±4.09		22.88±4.98		55.18±8.74	
Upper than bachelor	26.39±3.34		21.43±5.61		51.47±10.59	
Job State		0.215		0.091		**0.03**
Employed	26.68±4.01		23.21±5.54		55.66±10.31	
Unemployed	26±3.69		21.95±4.83		51.6±8.76	
Treatment		0.841		0.434		0.632
No treatment Rituximab IFN-B1a Fingolimod Ocrelizumab DMF Glatiramer acetate IFN-B1b Fampridine Teriflunomide	26.3±2.31 26.13±4.04 26.9±5.11 25.07±1.75 24.5±4.35 26.05±2.71 26.5±0.57 26.25±3.37 28.5±3.69 26.78±3.91		22.1±5.15 22.27±5.56 22.06±5.65 20.76±3.53 20.5±4.35 22.66±4.37 27.75±4.64 22.75±5.82 23±4.39 23.96±4.98		53.8±7.98 52.55±9.38 51.69±10.8 50.84±5.69 53.75±4.64 55.61±9.17 56.25±4.57 56.62±8.97 56.5±14.36 53.55±9.73	

DMF: Dimethyl fumarate, IFN: Interferon. Significant *p-value* is in bold

### Correlation between stigma score, MSIS-29 subscales, and clinical characteristics

Our findings demonstrated that stigma was significantly associated with MSIS-29-PSYCH (r = 0.468, *p-value* < 0.001) and MSIS-29-PHYS (r = 0.585, *p-value* < 0.001). Moreover, there was a significant positive correlation between MSIS-29-PHYS and MSIS-29-PHYS (r = 0.536, *p-value* < 0.001). More details on the correlation between stigma score, MSIS-29 subscales, and clinical characteristics are summarized in Table 4.

**Table 4:** Correlation between stigma score, MSIS-29 subscales, and clinical characteristics

Variables	Stigma		Psychological effect		Physical effect		EDSS		Disease duration		Treatment duration	
	r	*p*	r	*p*	r	*p*	r	*p*	r	*p*	r	*p*
Stigma	-	-	0.468	**<0.001**	0.585	**<0.001**	-0.074	0.308	-0.045	0.535	0.129	0.074
Psychological effect	0.468	**<0.001**	-	-	0.536	**<0.001**	-0.097	0.181	-0.075	0.301	0.005	0.944
Physical effect	0.585	**<0.001**	0.536	**<0.001**	-	-	-0.14	0.052	-0.162	**0.024**	0.061	0.4
EDSS	-0.074	0.308	-0.097	0.181	-0.14	0.052	-	-	0.173	0.17	0.018	0.804
Disease duration	-0.045	0.535	-0.075	0.301	-0.162	**0.024**	0.173	0.17	-	-	0.343	**<0.001**
Treatment duration	0.129	0.074	0.005	0.944	0.061	0.4	0.018	0.804	0.343	**<0.001**	-	-

DSS: Expanded Disability Status Scale, MSIS-29: Multiple Sclerosis Impact Scale-29. Significant *p-value* is in bold.

## Discussion

PwMS encounter alterations in their lives as a result of their medical condition. These alterations can include experiences of stigma [13]. Stigma can be defined as a negative perception towards chronically ill patients by their relatives, society, or the patients themselves [24]. The present study supports using the Persian version of RSS-MS, validating its internal structure as a one-factor solution with strong factor loadings. This indicates that our approach to measuring the feeling of stigma in pwMS was most accurately represented by a single construct, as each item in the scale contributed significantly to this construct. The nine items of the RSS-MS displayed excellent internal consistency, and removing low-total correlation items would not have enhanced Cronbach’s alphas. Consequently, each item in the Persian version of RSS-MS measure was valuable and informative in understanding the stigma of pwMS.

Stigma continues to be a significant concern for pwMS [25]. The rate at which pwMS disclosed having encountered stigmatization was found to be approximately 52.6% [26], 57.3% [27], and 79.2% [28] in different studies from different cultures, which highlights stigma presence as a prevalent problem among pwMS. PwMS may face concerns about anticipated stigma, leading them to feel compelled to conceal their diagnosis. This concealment may involve masking physical symptoms or utilizing assistive devices less frequently. Additionally, they may withdraw from sources of social support and isolate themselves, all with the intention of avoiding discussing their diagnosis due to anticipated negative appraisal [29]. Many pwMS conceal their condition due to social stigma, aiming to preserve their employment, social connections, and fear of the reactions and behaviors of society [30]. The stigma associated with chronic illnesses has a negative impact on the help-seeking attitudes of pwMS who exhibit elevated depressive and anxiety symptoms [31]. A large-scale national survey revealed that the longer individuals had lived with multiple sclerosis, the less stigma they experienced [32]. Previous studies showed that disability, cognitive decline, depression, anxiety, DMTs, psychiatric or nonpsychiatric illnesses, and employment status might potentially impact the severity of perceived stigma in pwMS [29, 33–36].

Our study revealed that stigma had significant associations with physical and psychological well-being. The experience of being stigmatized can have adverse effects on a person’s job performance and is linked to feeling restricted by physical or mental health issues, facing challenges in the workplace, and being unemployed [37]. Additionally, we showed that no difference was observed in stigma and psychological effects between employed and unemployed patients. However, another study documented that employed patients experienced a higher level of stigma, similar to those who are single, widowed, divorced, or separated [10]. A systematic review revealed that A considerable number of pwMS encounter stigma and discrimination in the workplace, and a significant number of them elect to refrain from disclosing their illness to their colleagues [38].

Based on our findings, addressing the stigmatization of pwMS is associated with many clinical benefits. This can lead to better medical outcomes, such as treatment adherence, and improved psychological well-being by addressing depression and anxiety. Ultimately, this could positively impact the patient’s overall QoL. Additionally, our findings show that stigma experience is unrelated to EDSS score or disease duration. While some of the previous studies found a significant relationship between stigma and disability in MS [32, 39]. However, physical effect is significantly and negatively correlated with disease duration, and there is also a negative correlation, but not significant, between EDSS score and physical effect. These findings are coupled with the prior findings of the relationship between disease course, the severity of the disease, and relapses with physical and psychological QoL [40]. In this regard, it was shown that an aggressive disease course is inversely correlated with QoL, and the rapid development of MS in a progressive course of MS is associated with poorer QoL [41]. Therefore, among different types of MS, relapsing-remitting MS had superior psychological and physical dimensions compared to the progressive types of MS [42].

Previous studies have noted that pwMS experienced moderate to severe stigmatization and subsequently lower overall QoL. It was shown that patients with work productivity ages are prone to experience productivity loss and require informal care associated with stigmatization and lower QoL [43]. The relation between a sense of coherence and perception of health was investigated in different diseases, and it is evident that a sense of coherence is a source of upholding health, particularly mental health, and leads to resilience [44]. Stigmatization of pwMS is a great source of depression, and patients with stigma are more disposed to symptomatic depression and getting clinical levels of depression [45].

The social support provided by a close relative has a substantial and profound effect on the patient’s perception of their purpose in life and overall contentment with life [46, 47]. Broersma et al. conducted the first study on the sense of coherence with stigma among pwMS [48]. It was shown that there is a connection between feeling stigmatized, having a sense of coherence, and experiencing limitations in patients’ QoL. Patients with higher limits, less coherence, and more stigmatization tend to have poorer physical and psychological health, social relationships, and environmental factors. Similarly, our findings also showed that stigma is closely correlated with physical and mental health issues impacting their QoL. Although disability of patients is the cornerstone predictor of QoL, some studies have also tried to investigate the putative predictors of the stigma of pwMS. Anagnostouli et al. [49] used Multiple-Sclerosis-QoL-54 (MSQoL-54) and Stigma-Scale-for-Chroic-Illness-24 (SSCI-24) for evaluating the disease-related variables and found that disability level, as the most strong variable, and also mental disease is predictors of stigma.

While some pwMS experience stigma, most patients experience at least some level of stigma, and some even experience a significant amount. MS is already an unpredictable and variable disease. It becomes even more challenging to understand when combined with an unknown subtype. This lack of understanding often increases stigma for those affected [50]. Therefore, patients try to conceal and suppress undesirable society-derived apprehension that makes patients isolated from society. It was noted that concealment is present in nearly 20% of patients, and previous studies have noted that concealment of patients can result in avoiding regular doctor appointments and treatment adherence. So, it can explain why nearly 10% of patients with MS have not been using disease-modifying therapies [51]. The findings of the Eldridge-Smith et al. [10] study suggest that a majority of pwMS reported experiencing differential treatment at some point due to their MS diagnosis. Also, sensing stigma can influence patients’ adherence to disease-modifying therapies. Cook et al. [52] noted a relation between concealment and adjournment of medical treatment. Considering that disease-modifying therapies are associated with the prevention of brain atrophy and the development of new lesions [12, 13], concealment and, subsequently, delay in starting medical therapies can lead to medically long-lasting damage and burden to the health community, patients, and their families, and governments.

### Strengths and limitations

Our study faces some drawbacks in the design and conduction. This is a cross-sectional study with a small sample size of pwMS in the Persian population that has never been studied before. Therefore, a longitudinal study with a large number of patients is required to conclude with absolute certainty. Second, all patients were recruited from the MS clinic of Isfahan University of Medical Science, Isfahan, Iran. Therefore, this single-center approach would be improved via a multi-center design to prevent any possible interactions between cultural differences and level of stigmatization. Previous studies have reported the different variables as predictors of stigma and QoL in pwMS, but no studies have conducted a comprehensive review on the predictors of stigma. Our study also tried to investigate the effect of the type of treatment and job state that had not been explored in previous studies.

## Conclusion

The finding of this study demonstrated that the Persian version of the RSS-MS scale is a reliable and validated tool for quantifying the stigmatization of pwMS. Moreover, the experience of stigma is significantly correlated with psychological and physical dimensions. There is potential use for this scale in clinical practice of MS to understand stigmatization, and it may be effective in treatment planning and prognosis of the disease. The adult long form of the RSS-MS scale exhibited high internal consistency and is suitable for the Persian population.

### Electronic supplementary material

Below is the link to the electronic supplementary material.

Supplementary Material 1

## Data Availability

The data supporting the findings of this study is available from the corresponding author upon reasonable request.

## Abbreviations

CNS: Central nervous system
CVI: Content validity index
CVR: Content validity ratio
EDSS: Expanded Disability Status Scale
FA: Factor analysis
GBD: Global Burden Disease
IFN-B1a: Interferon-B1a
KMO: Kaiser-Meyer-Olkin
ML: Maximum likelihood
MSIS-29: Multiple Sclerosis Impact Scale-29
MSIS-29-PSYCH: 29-item scale measuring psychological influence
MS: Multiple sclerosis
PwMS: People with multiple sclerosis
Qol: Quality of their lives
RSS-MS: Reece Stigma Scale Multiple Sclerosis
SD: Standard deviation
