# Supplementary material for: Validity and reliability of the Persian version of Recce stigma scale in people with multiple sclerosis and its impact on quality of life
Source: BMC Neurol. 2024 Jan 22;24:36. doi: 10.1186/s12883-024-03544-z (PMC10802045; doi:10.1186/s12883-024-03544-z)
Supplement: Supplementary file 1 — Supplementary Material 1 [file 12883_2024_3544_MOESM1_ESM.docx]

Table S1. The Reece Stigma Scale Multiple Sclerosis (RSS-MS) (1) questionnaire was used for Persian patients with multiple sclerosis.

| In the past 30 days, how often have the following statements applied to you: | Never | Rarely | Sometimes | Often | Always |
| --- | --- | --- | --- | --- | --- |
| 1. Felt that having MS was a punishment for things I had done in the past. |  |  |  |  |  |
| 2. Felt that people were avoiding me because of my MS. |  |  |  |  |  |
| 3. Feared that I would lose my friends if they found out about having MS. |  |  |  |  |  |
| 4. Felt like people that I know were treating me differently because of my MS. |  |  |  |  |  |
| 5. Felt like people look down on me because I have MS. |  |  |  |  |  |
| 6. Avoided dating because most people don’t want a relationship with someone with MS. |  |  |  |  |  |
| 7. Avoided a situation because I was worried about people knowing I have MS. |  |  |  |  |  |
| 8. Was embarrassed about having MS. |  |  |  |  |  |
| 9. Felt that keeping my MS a secret was important. |  |  |  |  |  |
|  |  |  |  |  |  |

MS: Multiple sclerosis.

1. Eldridge-Smith ED, Loew M, Stepleman LM. The adaptation and validation of a stigma measure for individuals with multiple sclerosis. Disabil Rehabil. 2021;43(2):262-9.
